# Where Sounds Occur Matters: Context Effects Influence Processing of Salient Vocalisations

**DOI:** 10.3390/brainsci10070429

**Published:** 2020-07-06

**Authors:** Atiqah Azhari, Paola Rigo, Marc H. Bornstein, Gianluca Esposito

**Affiliations:** 1 Psychology Program, School of Social Sciences, Nanyang Technological University, Singapore 639798, Singapore; nura0066@e.ntu.edu.sg (A.A.); paola.rigo.1@unipd.it (P.R.); 2 Department of Developmental and Social Psychology, University of Padova, 35131 Padova, Italy; 3 Eunice Kennedy Shriver National Institute of Child Health and Human Development, NIH, Bethesda, MD 20817, USA; marc.h.bornstein@gmail.com; 4 Institute for Fiscal Studies, London WC1E 7AE, UK; 5 Department of Psychology and Cognitive Science, University of Trento, 38068 Trento, Italy; 6 Lee Kong Chian School of Medicine, Nanyang Technological University, Singapore 639798, Singapore

**Keywords:** emotional sounds, context effect, emotion regulation, prefrontal cortex, fNIRS

## Abstract

The social context in which a salient human vocalisation is heard shapes the affective information it conveys. However, few studies have investigated how visual contextual cues lead to differential processing of such vocalisations. The prefrontal cortex (PFC) is implicated in processing of contextual information and evaluation of saliency of vocalisations. Using functional Near-Infrared Spectroscopy (fNIRS), we investigated PFC responses of young adults (N = 18) to emotive infant and adult vocalisations while they passively viewed the scenes of two categories of environmental contexts: a domestic environment (DE) and an outdoors environment (OE). Compared to a home setting (DE) which is associated with a fixed mental representation (e.g., expect seeing a living room in a typical house), the outdoor setting (OE) is more variable and less predictable, thus might demand greater processing effort. From our previous study in Azhari et al. (2018) that employed the same experimental paradigm, the OE context was found to elicit greater physiological arousal compared to the DE context. Similarly, we hypothesised that greater PFC activation will be observed when salient vocalisations are paired with the OE compared to the DE condition. Our finding supported this hypothesis: the left rostrolateral PFC, an area of the brain that facilitates relational integration, exhibited greater activation in the OE than DE condition which suggests that greater cognitive resources are required to process outdoor situational information together with salient vocalisations. The result from this study bears relevance in deepening our understanding of how contextual information differentially modulates the processing of salient vocalisations.

## 1. Introduction

From laughter to cries, the human experience is characterised by a plethora of emotional vocalisations of varying affective prosody that serve to convey a wealth of information [1,2]. Classical theories of emotions are founded on the belief that vocalisations relay discrete universally recognised emotions [3,4]. Cries are commonly thought to automatically invoke sadness, and laughter to excite joy [5]. However, the realisation that vocalisations rarely occur in a social void has led to an emerging understanding of how contexts moderate the interpretation of emotional vocalisations. Indeed, context serves as a framing backdrop against which we derive situationally relevant meaning from our sensory experiences [6].

The social context network model (SCNM) suggests that a neural network which comprises the frontal, insular and temporal regions of the brain is responsible for social cognition processes related to context [7]. Based on this proposition, the SCNM taps upon contextual information to predict events that are about to happen, and integrate context-target mental connections [7]. Several past studies have proved that contextual targets are predicted and updated based on existing information and retrieval of relevant episodic memories [8,9]. Environments in which unpredictable events occur elicit anxiety-like behaviours and lead to a heightened state of amygdala habituation in humans [10]. In fact, unpredictable contexts have been shown to induce observable cerebral changes, such as enhancing cognitive processing of a stimulus so as to rapidly evaluate its significance [11]. Similarly, context lends interpretation to salient emotional vocalisations. The functional-contextual theory of emotions posits that vocalisations only serve to attract attention and are subsequently interpreted in a context-dependent manner [12,13,14,15,16]. Recently, ref. [17] demonstrated the perceptually ambiguous nature of human vocalisations, and how reliant vocalisations are on contextual information to elicit emotional reactions in listeners. An environment that is unfamiliar (e.g., walking in an alley), for example, may lend a sinister interpretation to laughter compared to when laughter is heard in an environment where the vocalisation is deemed ordinary (e.g., comedy show). Thus, the type of environmental context that a salient vocalisation is presented with may serve a prime function in influencing its interpretation. The prefrontal cortex (PFC) is a hub in which both the processing of salient sounds and context-dependent evaluation of social cues, occur [18]. Upon exposure to vocalisations, accumulating evidence points to the initial involvement of the amygdala and the temporal lobe during early processing of affect [19,20] followed by higher-order evaluative analysis in distinct areas in the PFC [21,22,23,24,25]. Evaluation of sounds together with contextual information also falls within the purview of the PFC. In an fMRI study which showed the context-dependent nature of sound processing, [26] used 640 images from four scene categories (beach, office, city, forest) and 64 sound clips that corresponded to the same scenes (e.g., sound of waves on beach, people talking in the office) to discover that, when the two modalities are congruent to the same scene category, a similar neural activation pattern in response to visual and auditory stimuli occurs only in the PFC, suggesting its critical role in coordinating abstract visual and auditory scenes relative to context. Neural activation patterns were found to be distinct in different categories of scenes, indicating that contextual interpretations of scenes may fall within the purview of the PFC. Taken together, these findings point to the singular function of the PFC in the processing of both salient sounds and contextual information.

Despite these extensive findings, no study to date has investigated the cerebral manifestation of processing salient human vocalisations in contexts which differ in familiarity. Given the emergence of recent findings which support the notion of a contextually derived meaning of human vocalisations [17], emotional processing of vocalisations might likewise be modulated by the familiarity of contextual information to elicit specific differences in the brain. Such an investigation would be significant in lending insight into how individuals might perceive and respond to vocalisations differently across various environments. On this basis, we proposed to embark on an exploratory investigation of whether a more familiar domestic (DE) environment, compared to a less familiar outdoor environment (OE) moderate PFC responses to salient cry and laughter vocalisations differently. Compared to the domestic context which is accompanied by a fixed mental representation (e.g., anticipate seeing a living room in a typical domestic setting), the outdoor context depicts unfamiliar and more variable visual information that is less comparable to individuals’ previous experiences. Thus, these two categories of contextual cues could differentially influence emotional processing of salient vocalisations.

Using the same experimental paradigm, our previous study [27] investigated how DE and OE scene categories affected the physiological arousal of men and women when presented with salient vocalisations. Physiological arousal is an involuntary increase in activity of the sympathetic branch of the peripheral nervous system (PNS), which is responsible for a “fight or flight” response typically triggered by stressful situations. Unpredictable stimuli have indeed been shown to increase sympathetic arousal [28]. Our prior study [27] found that greater arousal in the OE compared to the DE condition was observed in women. This observation could be due to the pairing of unpredictable outdoor contextual scenes with salient vocalisations which warranted an increase in recruitment of processing resources [29], that was reflected in enhanced physiological arousal.

The present follow-up study to [27] explores PFC activity in listeners in response to vocalisations presented in domestic (DE) and outdoor (OE) environments. In the previous study, [27] used both a passive and an active task to account for differences in cognitive engagement with contextual cues. As the present study is a preliminary and exploratory investigation, only the passive task, where participants are instructed to view visual scenes which are presented to them, is included. We embarked on this study with one central hypothesis. Mirroring the findings from the previous study [27], we expected OE, compared to DE, to demand greater attentional resources that would be accompanied by an increase in brain activity to accommodate greater processing resources demanded of an unfamiliar setting.

## 2. Materials and Methods

### 2.1. Participants

We recruited a total of 39 nulliparous Asian Chinese ethnic young adults for this study (12 females, M age = 22.0 ± 1.87 years; 27 males, M age = 22.3 ± 2.0 years). Inclusion criteria were to be non-parents, 18 to 30 years of age and without a personal or family history of neurological or mental disorders. However, after applying a conservative signal quality check, data from only 18 participants (5 females, M age = 20.4 ± 1.14 years; 13 males, M age = 23 ± 1.87 years) were eventually included for analyses. All participants were Psychology undergraduate students who were recruited through the university’s online portal. They received academic credits for their participation. Participants gave informed consent to participate, and the study was approved by the ethical committee of the Psychology Division (Protocol ID 09/07/2016), Nanyang Technological University, Singapore. All data are available at this URL: https://doi.org/10.21979/N9/SJYOIB.

### 2.2. Visual Stimuli

**Figure 1:**
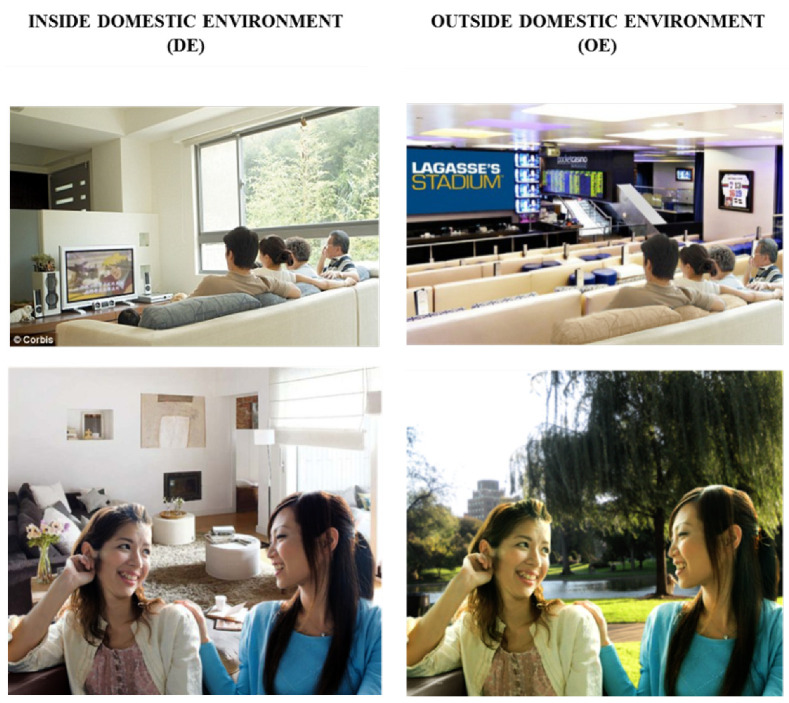
Visual stimuli. Two examples of visual scenes used in the study (actions: above—watching television; below—conversation) in domestic environment (DE) and outdoors environment (OE) conditions. The same person(s) were included in both DE and OE backgrounds.

Visual stimuli consisted of 4 different images within a domestic environment (DE; domestic kitchen, entrance of an apartment, living room and television room), and 4 different images from an outside environment (OE; commercial kitchen, bus stop, public television lounge and park) (Figure 1). The background images were first sourced and obtained from a public online platform using Google Images Search Engine. We performed an advanced search for images, which were filtered based on whether they were free to use, share, or modify commercially. Foreground images of persons performing 4 different common actions in daily life (cooking, texting on the phone, watching the television and chatting) were obtained in the same manner. Each foreground image was transposed onto a background image from the DE category and a different background image from the OE category (e.g., two women chatting (foreground image) was depicted in a living room (DE background) and at the park (OE background)). Persons performing specific actions in the foreground image were of Asian Chinese ethnicity to reflect the sample tested. Foreground images were transposed to corresponding background images using Adobe Photoshop CC (www.adobe.com/sea/products/photoshop.html), and all images were further processed to equate for brightness and resolution.

Prior to the experiments, a pilot study was conducted on 21 adult participants (Mean age = 27; SD = 8.0). The questionnaire asked participants to rate the familiarity of the 8 contextual scenes on a Likert scale from 0–10, where 0 denoted no familiarity at all and 10 denoted complete familiarity. For example, the question on familiarity of a commercial kitchen was: “A commercial kitchen (like the one pictured below) is a familiar place to me”. The image of the commercial kitchen that was used as a stimulus was displayed to the participant at the same time. Contextual scenes from the DE category were consistently rated as more familiar than the scenes in the OE category across both samples. The overall familiarity score of DE was 8.1 (SD = 2.2) whereas that of OE was 5.6 (SD = 3.6), Two-sided paired *t*-test analysis between DE and OE conditions generated a significantly greater familiarity score in DE compared to OE (t = 5.60, df = 83, *p* = 2.75 × 10${}^{-7}$). The pilot study showed that adults generally find scenes from the domestic environment more familiar compared to the non-domestic environment, when the actions within these scenes were controlled for.

### 2.3. Auditory Stimuli

Four sound categories consisted of 4 items each: infant cries (IC; hunger cries from 1-year-old infants), infant laughs (IL; from 1-to 2-year-olds), female adult cries (AC) and control sounds (CS). Human social vocalisations (i.e., IC, IL, AC) were retrieved from Oxford Vocal (OxVoc) Sounds database (Parsons et al., 2014) and public online databases (www.sounddogs.com;www.soundbible.com; www.audio4fun.com; https://www.freesound.org). For the present study, 15 s of the original IC and IL files were excerpted (sampling rates 44.1 kHz/32 bit), background noise was removed and sounds were matched for volume using Audacity 2.0.4 (http://audacity.sourceforge.net). To control for the acoustic structure of infant cries [30,31], CSs that mimicked the temporal pattern of ICs were generated using Cool Edit Pro Version 1.2 Syntrillium Software (http://www.syntrillium.com).

### 2.4. Experimental Design

To examine different context effects, each participant underwent two experimental sessions: DE and OE. In the DE condition the images were only from the DE context, and in the OE condition the images were only from the OE context (Figure 1). The effects of sound were investigated in each session, such that all 4 sounds (IC, IL, AC, CS) were presented in both DE and OE conditions. To simulate real-life events where adults are commonly engaged in activities before hearing salient cues, participants were not instructed to attend to sound stimuli explicitly. The stimuli and experimental design were previously used in [27] where physiological activation through electrocardiogram (ECG) was measured.

### 2.5. Procedure

**Figure 2:**
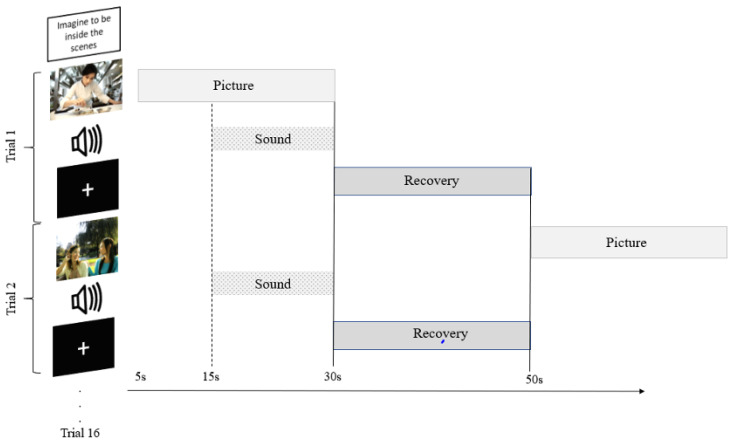
Experimental design. Schematic diagram illustrating onset and offset of picture and sound pairings for each trial.

Before the experiment, each participant was randomly assigned to the domestic environment (DE) or outdoor environment (OE) condition first so that conditions were counterbalanced across participants. The instruction “look at the scenes” was displayed at the start of the experiment for both the DE and OE conditions. Each experimental session consisted of 16 trials. For each trial, one image was presented for a total duration of 25 s. The onset of the auditory stimulus occurred after the image had been presented for 10 s, accompanying the last 15 s of the visual stimulus (Figure 2). The offset of both the image and sound was followed by a fixation point, which was displayed at the centre of the blank screen. A recovery period lasted for 20 s before the subsequent trial commenced. The order of the sound stimuli as well as the image-sound pairing in each trial were randomised.

### 2.6. Functional Near-Infrared Spectroscopy (fNIRS) Data Acquisition

The functional Near-Infrared Spectroscopy (fNIRS) neuroimaging system (NIRSport, NIRx Medical Technologies LLC) was used to measure the dual-wavelength signal changes (850 and 760 nm), corresponding to levels of oxygenated haemoglobin (OxyHB) in the PFC. Optical signals were collected using fNIRS optodes connected to the head cap of 8 LED sources and 8 detectors. The optode holders in the fNIRS cap were placed in accordance with the standard 10-5 positions, with a recommended maximum of 3 cm spacing between each probe (http://nirx.net/nirscaps/). NIRStar software (NIRx Medical Technologies LLC, Berlin, Germany) was used to configure a 20-channel-recording system of the PFC, utilising an 8 × 7 source-detector montage. The distance between sources and detectors did not exceed 3 cm (optimal interoptode distance). The signal was recorded at a sampling rate of 7.81 Hz. The fNIRS allowed monitoring local blood oxygenation, and more active brain areas exhibited greater concentrations of oxygenated haemoglobin (OxyHB) relative to deoxygenated haemoglobin (deoxyHB). At the start of each experimental session, the probes on the fNIRS cap were adjusted before calibration of signal quality. A preview of the fNIRS data recording was performed before the actual recording using NIRStar system control software. NIRStim software (NIRx Medical Technologies LLC, Berlin, Germany) was used to present the visual and auditory stimuli for both experiments. At the end of the first experimental session, recording was stopped and the fNIRS signal was recalibrated before continuing with the second experimental session.

### 2.7. fNIRS Pre-Processing and Analyses

Pre-processing and analysis of fNIRS data were conducted using NIRSlab software [32,33]. Channel exclusion was determined to be excessively noisy upon visual inspection with NIRS v.205 software (NIRx Medical Technologies LLC, Berlin, Germany), such that channels with significant background noise (gain > 8, Coefficient of Variation (CV) > 7.5) were excluded. Datafiles with fewer than 10 acceptable channels were also omitted. Markers for onset of auditory stimuli were added. Discontinuities were removed, and spikes were replaced with signals nearest to the spike artefacts. Before haemodynamic states were calculated, a band-pass filter was applied (0.1–0.2 Hz) to remove any physiological slow signals and baseline shift variations. The pre-processed optical signals were converted to concentration changes in OxyHB and DeoxyHB using the modified Beer–Lambert law for each channel. Two levels of analyses were conducted for fNIRS data: within-subject analysis (first-level) and group-level analysis (second-level). First-level analysis was specified as a haemodynamic response function (HRF), and pre-whitening was omitted. A convolution design matrix plotted the 4 sound conditions against a time axis, at which point the matrix was checked against the order of stimulus presentation which the participant received. A DCT temporal parameter with a high-pass period cut-off of 128 secs was applied. The analysis followed a Gaussian FWHM (i.e. full width at half maximum 4 model, and for each individual participant general linear models (GLM), from OxyHB and DeoxyHB signals, were estimated to obtain the beta-coefficient for each of the four sound conditions. At group-level analysis, beta-coefficients from OxyHB GLM of each participant were aggregated into a group-level GLM. We implemented an analysis of variance (ANOVA) analysis with two repeated measures (within subjects’ effects) for context effects (DE, OE) with two levels, and sound effects (IC, IL, AC, CS) with 4 levels. The participant’s sex was a covariate in the model. This analysis was conducted on all 20 channels and results were subjected to Bonferroni correction (*p* < 0.05) and false discovery rate (FDR) correction to account for multiple comparisons across the 20 channels. Results were depicted in a topographical map with probe labels to allow for subsequent mapping of brain regions. To verify the HRF with respect to the DeoxyHB signal, the same level group analysis was performed on the beta-coefficients derived from the DeoxyHB signal.

## 3. Results

To test whether context is associated with different PFC activation, a two-way repeated-measures analysis of variance (ANOVA) was conducted. Analyses revealed a main effect of context in CH11 (F(1,16) = 21.3, *p* = 0.000287, *p*-corrected = 0.00485, ηp2 = 0.571) that remained significant after FDR correction for comparisons across multiple channels. This channel mapped to the left rostrolateral PFC (RLPFC) in BA10. No significant effect of sound emerged.

**Figure 3:**
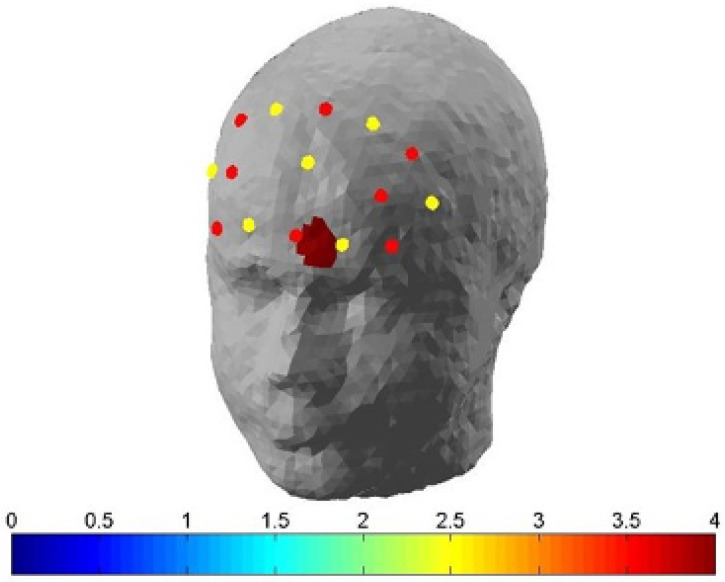
Topographical map of outside of domestic environment (OE) condition–domestic environment (DE) contrast. Topographical map depicting higher OxyHB concentration in channel 11 during response to sounds in the OE, as compared to the DE condition. This channel was located in the left rostrolateral prefrontal cortex.

To test the hypothesis that OE is associated with greater activation of the PFC compared to DE, a pairwise t-test analysis was performed. Pairwise comparisons showed greater cerebral activation to sounds in the OE as compared to the DE condition in CH11, which corresponds to the left RLPFC (CIlower = 0.000086, CIupper = 0.000270, *p*= 0.0008; Figure 3).

Analyses of repeated-measures ANOVA were also conducted on changes of DeoxyHB concentrations. No significant findings emerged.

## 4. Discussion

This study preliminarily investigated how different contexts influence the activation of the prefrontal cortex (PFC) when processing salient emotive vocalisations. We tested the hypothesis that contextual scenes that depicted the outdoor environment (OE) would elicit greater activation of the PFC compared to scenes of the domestic environment (DE), when paired with salient vocalisations. This hypothesis was fulfilled as we found that the left rostrolateral PFC, which is associated with higher-order processing and relational integration, was more activated in the OE condition. An effect of context in this brain area suggests that processing of salient vocalisations which occurred in the less familiar outdoor context, compared to the more familiar domestic context, recruited greater cognitive resources for the integrated processing of multiple visual and auditory relations [34,35].

The finding that a less familiar outdoor environment elicited greater cognitive response is in line with the available literature which suggests that uncertain contextual settings demand greater recruitment of processing resources [29,36]. From an evolutionary standpoint, the human’s capacity to formulate consistencies and rapidly extricate irregularities from our surroundings is essential to our survival. When presented with a social context, humans depend on our past mental representations of a similar social scene to anticipate the upcoming events that are expected to occur within that context [37]. These frameworks that we have come to rely upon allows us to quickly identify unexpected changes in our social environment [38]. Compared to the domestic context, the outdoor context is less familiar and possesses more variable mental representations. For instance, a visual depiction of two women chatting on a bench offers little information as to what to expect from the women’s surroundings. By contrast, two women chatting on a living room sofa immediately calls to mind well-learned mental representations of a household setting. Thus, information processing of OE visual stimuli could have demanded greater processing resources compared to that of DE that contributed to greater cognitive activity observed in the OE condition.

With the limited attentional capacity that we possess, processing of the less familiar outdoor scenes could have competed with the cognitive mechanisms required to process salient vocalisations [39]. The brain area that emerged to be implicated in the simultaneous competition for processing of both visual and salient auditory stimuli is the left rostrolateral PFC. This region is known to be recruited for relational integration and reasoning, that is, the process of considering multiple available relations simultaneously [34,35]. Several studies have ascertained that emotional recognition of vocalisations demands attentional [40] and prediction resources [37]. Furthermore, human vocalisations which are salient and convey the emotional state of another person are automatically prioritised when pitted against other competing stimuli for selective attention [15,16,36,39,41]. Therefore, integrating salient vocalisations with the ambiguous and unfamiliar OE contextual scenes could have demanded greater recruitment of the rostrolateral PFC so as to make meaning of both the auditory and visual stimuli.

Our findings support the notion that the cognitive response towards salient vocalisations is contingent upon the contextual environment which the vocalisations are paired with. These results corroborate with that of [27] in which greater sympathetic arousal and lower heart rate variability was found in the OE condition. Similarly, the present study demonstrated that young adults exhibited greater cerebral activation towards vocalisations in the OE condition. Taken together, results from the two studies suggest that contextual cues of the less familiar outdoor environment demanded greater recruitment of processing resources in response to emotive vocalisations at the levels of both the brain and sympathetic nervous system. Indeed, numerous studies have revealed the interconnectedness of these two systems [42]. Of particular relevance to the present discussion is the association between higher physiological arousal with less efficient cognitive processing involving executive functioning [43,44,45]. Based on this prior established functional relation, it appears that findings from both the present study and the previous one [27] point to the enhanced cognitive processing demanded of the OE condition.

It must be stressed that this study is a preliminary and exploratory investigation of the differential effects of context on the processing of salient vocalisations. Hence, it has several limitations that should be addressed. First, the small sample size may subject the analyses to increased alpha and beta errors. Replication studies for this experiment is required on a larger sample size to mitigate this limitation and confirm the findings. Second, this study did not investigate sex-based differences in participants. Past studies have attested to sex differences in processing of emotional speech and emotional prosody of vocalisations that are reflected in the brain [46,47]. Moreover, across both auditory and visual modalities, women have consistently been shown to be more adept at emotional recognition compared to men [48,49,50,51]. A future expansion to this study would be to consider the effects of the sex of the listener when investigating contextual moderation in relation to vocalisations. Third, the contextual information provided to participants appeared in the form of visual scenes. In reality, the environment is multidimensional, comprising information from multiple modalities. It remains to be seen how auditory input of domestic (as compared to outside) settings might differently influence processing salient vocalisations. Given that the target stimuli are auditory in nature, auditory environmental input may prompt greater competition of cognitive resources. Fourth, as this study was conducted in Singapore, which is ranked as the 2nd safest city in the world in 2019 [52], on a Singaporean sample, outdoor scenes such as alleys are typically not construed as dangerous. However, there may be cultural differences in the perception of relative safety between indoor and outdoor contexts that could be further investigated. Fifth, this study focused on a specific region of the brain, the prefrontal cortex. As such, we were not able to capture neural mechanisms found in other parts of the brain (e.g., temporal and parietal lobes) which might otherwise offer a more comprehensive understanding of processing mechanisms in different contextual situations. Future research should address these limitations.

## 5. Conclusions

From an infant’s wail to an adult’s chortle, salient vocalisations serve as rich sources of social information that direct our behaviours in everyday life. This study uncovered the potential role of contextual information in eliciting different neurophysiological responses to vocalisations. We demonstrated that contextual moderation was evident during passive viewing of visual scenes, wherein more cognitive resources were required for processing of outdoor situational information together with emotive sound stimuli. This finding bears significant relevance in furthering our understanding of how the contextual environment that we are in influences the way we process salient social information.

## Abbreviations

The following abbreviations are used in this manuscript:

BA	Brodmann Area
CH	Channel
DE	Domestic Environment
DeoxyHB	Deoxyhemoglobin
DLPFC	Dorso Lateral Pre Frontal Cortex
fNIRS	functiona Near-InfraRed Spectroscopy
GLM	General Linear Model
HRF	Haemodynamic Response Function
OE	Outdoor Environment
OxyHB	Oxygenated Haemoglobin
PFC	Prefrontal Cortex

**Stimuli**

AC	Adult Cry
CS	Control Sounds
IC	Infant Cry
IL	Infant Laugh
